# Making from Breaking: Degradation Inversion Enables Vapor-Phase Synthesis of Halide Perovskites in Ambient Conditions

**DOI:** 10.1021/acsenergylett.4c03395

**Published:** 2025-05-12

**Authors:** Austin G. Kuba, Florent Sahli, Mostafa Othman, Kerem Artuk, Quentin Jeangros, Aïcha Hessler-Wyser, Christophe Ballif, Christian M. Wolff

**Affiliations:** † 27218 Ecole Polytechnique Fédérale de Lausanne (EPFL), Institute of Electrical and Microengineering (IEM), Photovoltaics and Thin-Film Electronics Laboratory, 2002 Neuchâtel, Switzerland; ‡ 122364 Centre Suisse d’Electronique et de Microtechnique (CSEM), 2002 Neuchâtel, Switzerland

## Abstract

Vapor phase deposition of formamidinium-rich perovskites is hampered by the decomposition of formamidine in the vapor phase. We use the reversibility of this reaction to establish a dynamic equilibrium that enables the vapor-phase synthesis of formamidinium iodide from the “degradation products” s-triazine, ammonia, and hydrogen iodide and thereby convert lead halide thin films to lead halide perovskites. Finally, we produce the first proof of concept solar cells via this innovative process.

## Graphical abstract

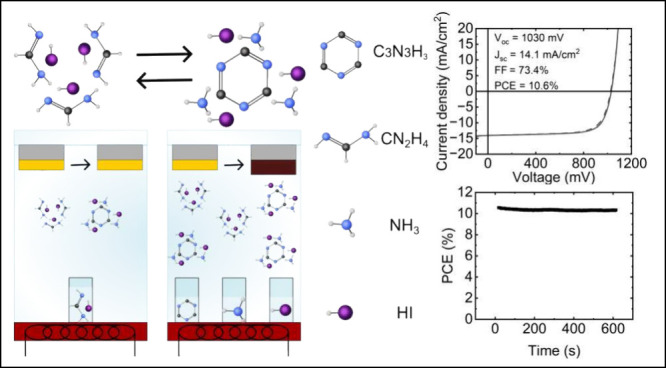

Perovskite solar cells have improved in recent years to nearly match traditional silicon solar cells in record small-scale solar cell efficiency.^1^ Although more work is necessary to ensure their success in real-world conditions, significant progress has also been accomplished that has increased their operational stability by orders of magnitude,^2–7^ leading to hopes of commercialization in the near future. In addition to insufficient reliability, a remaining challenge is the scalability and manufacturability of the fabrication techniques currently used to create perovskite solar cells.

Current record-setting small-scale devices are typically made through spin coating techniques.^8–10^ However, vapor-based processes have shown promising results in stability^11^ and scalability,^12,13^ and have recently made major strides in power conversion efficiency (*PCE*),^14–16^ nearly closing the gap with spin coated perovskites. Initial efforts focused on methylammonium lead iodide, which decomposes reversibly into PbI_2_, methylamine (MA) and HI^17,18^ making vapor processing reasonably straightforward and even allowing direct synthesis from delivery of MA and HI.^19^ However, due to thermal stability and performance benefits, current record spin coated and vapor processed device compositions are typically rich in formamidinium (FA).^8–10,14–16^ This often stymies efforts to develop high-rate vapor deposition of FA-rich perovskites, which is made difficult by the unstable nature of free formamidine.^20^ Evaporation of formamidinium is known to follow multiple decomposition pathways into several molecules, including HCN, ammonia, iodine, as well as a pathway where FA trimerizes into s-triazine with the release of NH_3_.^13,21–25^ Strikingly, the condensation reaction depicted in eq 1 that forms s-triazine from FA has been known since the 1950s to be used in the reverse direction to synthesize high purity amidines,^20,26^ showing this must be a reversible reaction.

$$C_{3}N_{3}H_{3}+3{\mathrm{NH}}_{3}⇌{\mathrm{CN}}_{2}H_{4}$$ 1

The “degradation” components are highly volatile and do not incorporate into the perovskite lattice.^23,24^ This leads to a new pathway for perovskite device manufacturing. In this work, we deliver highly volatile s-triazine, ammonia, and HI to a predeposited lead-halide-alkali-halide template film, which generates formamidine *in situ* in the reaction zone. This FA incorporates into the inorganic template with the HI to form a FA_*x*_Cs_1–*x*_Pb­(I_*y*_Br_1–*y*_)_3_ perovskite. We also demonstrate and characterize the first working solar cells made using this synthesis technique.

A schematic of the reaction process is shown in Figure 1. To make perovskite thin films, first, a lead halide template was produced by coevaporating CsBr (0.1 Å/s) with PbI_2_ (1 Å/s) for a total thickness of 30 nm CsBr and 300 nm PbI_2_. Next, different amounts of s-triazine, an ammonia source such as ammonium hydroxide (NH_4_OH) solution or ammonium acetate (NH_4_CH_3_CO_2_, abbreviated NH_4_oAC), and HI were placed in separate 4 mL vials. Next, a glass staining jar (Figure S1) and its lid were preheated on a hot plate at 150 °C. The lead halide template was taped to the lid and allowed to preheat for 5 min. The reactant vials were placed into the hot staining jar and the lid with the lead halide template was quickly placed on the jar. The reaction was allowed to proceed for a defined time period (typically 11–15 min during which most of the visible change in film color occurred), whereupon the lid was removed, the film was detached from the lid and annealed at 150 °C for 1 min. During the reaction process, the films turned dark brown to black to the eye. After each reaction the jar was left open on the hot plate and dried with N_2_ gas to reset the reaction environment. The fabrication was carried out in ambient atmosphere with uncontrolled room humidity (30–60%).

**Figure 1:**
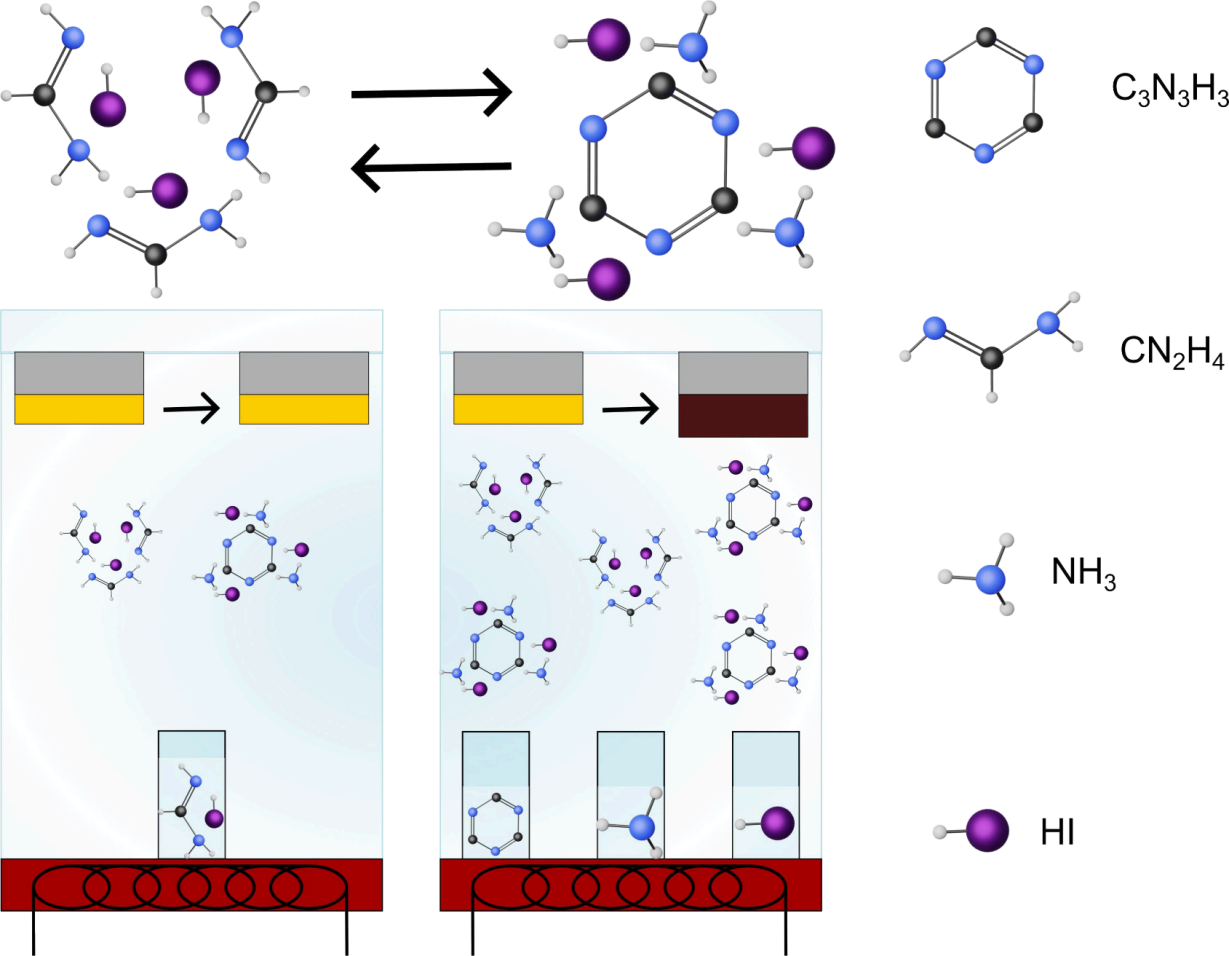
Schematic depiction of the reaction scheme. When using FAI as a precursor, the partial pressure is low and the FAI decomposes to components such as s-triazine, ammonia, and HI, hindering the reaction. When using s-triazine, ammonia, and HI, the components can be completely evaporated above the boiling point at room temperature, and the components react to form FAI, resulting in film conversion.

First, we sought to understand the chemical composition of the films produced by this process. ^1^H nuclear magnetic resonance (NMR) of six films reacted at the same conditions dissolved in dimethyl sulfoxide-*d*_6_ (DMSO-*d*_6_) confirms the presence of FA in the films (Figure 2a). S-triazine and ammonia did not incorporate into the films in detectable quantities, as evidenced by their absence in the NMR spectrum. ^1^H NMR spectra of s-triazine, HI, FAI, and NH_4_oAC are shown in Figure S2 for comparison. This confirms that the s-triazine and ammonia react to form formamidine in the vapor or on the surface of the film, and the FA incorporates while the s-triazine and ammonia do not. X-ray diffraction (XRD) was used to probe the crystalline structure of the film and the completeness of the reaction of the template into perovskite. An α-phase FAPbI_3_-perovskite type phase was identified in all films with brown to black appearance. An example diffraction pattern using optimized reaction conditions is given in Figure 2b. In all films, there were also detectable traces of δ-phase CsPbI_3_ and hexagonal polytypes of FAPbI_3_,^27,28^ and typically a residual PbI_2_ phase that varied in intensity with the amount of reactants added and conversion time.

**Figure 2:**
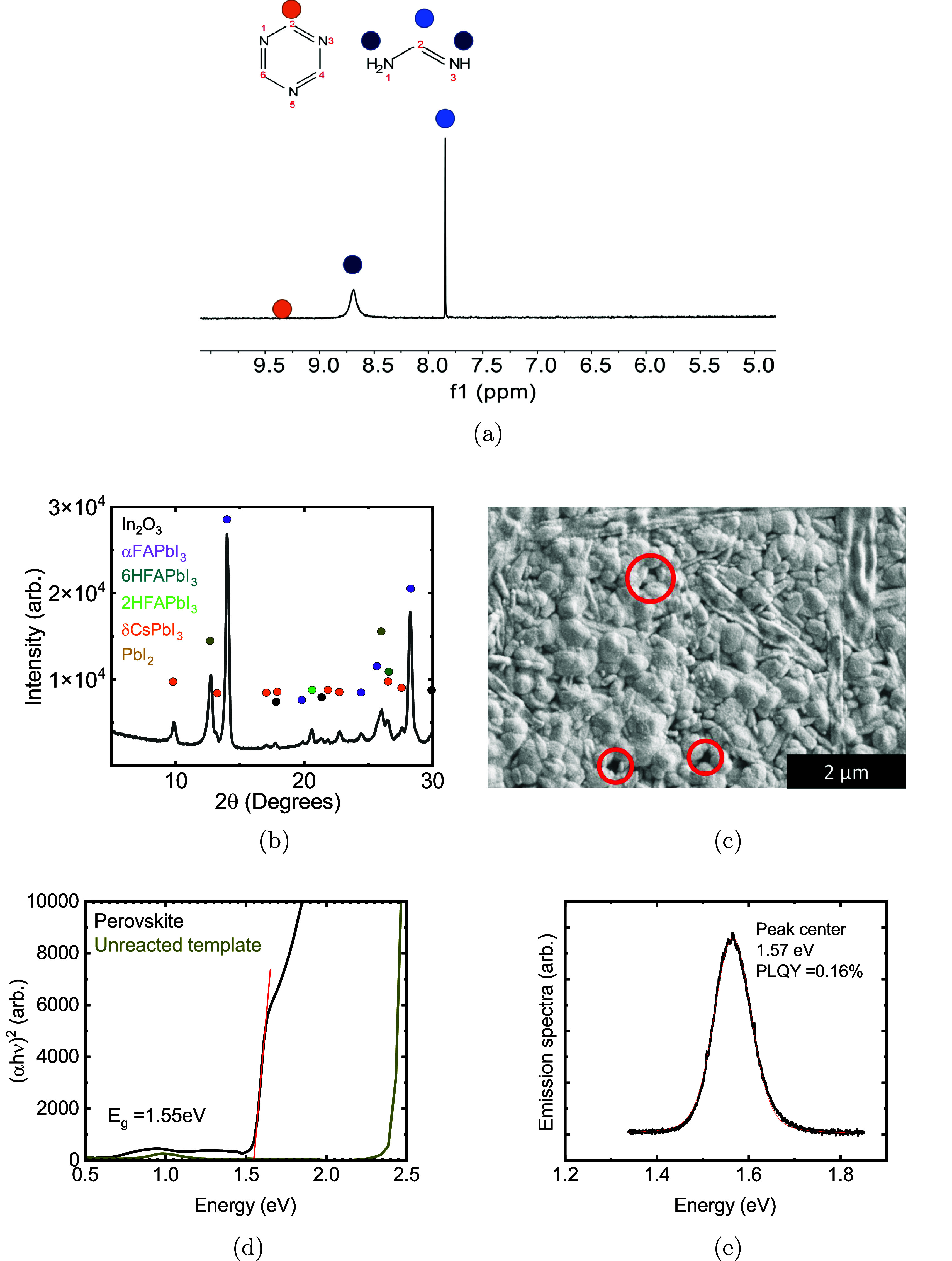
(a) ^1^H NMR of six perovskite films dissolved in DMSO-*d*_6_ showing FA incorporation with undetectable levels of s-triazine and ammonia. (b) X-ray diffraction pattern of a perovskite film showing significant α-phase perovskite with impurity phase inclusions. (c) SEM of a film with 100 μL of NH_4_OH addition showing relatively compact morphology with some needle-like features and pinholes (red circles). (d) A Tauc plot of a film with an optical bandgap of 1.55 eV. (e) A PLQY emission spectrum of the same film with PLQY = 0.16% and a peak center at 1.57 eV.

Next, the concentration of each reactant was varied to achieve films with nearly complete conversion, as measured by low-to-undetectable PbI_2_ signal in the X-ray diffractograms, and understand the effect of reactants on phase composition and morphology. It was found that increasing the concentration of each reactant increased the reaction rate (Figures S3–S6), albeit to different degrees. HI had a more significant effect on reaction rate than NH_4_OH and s-triazine within ∼2× variation in the addition of each reactant. Increasing the HI addition also correlated to an increase in peaks corresponding to hexagonal FAPbI_3_ polytype phases^28^ (Figure S5), showing that there is a trade-off between increasing reaction rate through increased HI concentration and the phase composition of the films. It was also possible to convert the films using solid I_2_ beads instead of HI, although films made in initial trials with HI appeared more uniform and reproducible (Figure S12) so HI was used for the remainder of the trials. Increasing the s-triazine did not have noticeable detrimental effects on phase composition or morphology but had a minor effect on reaction rate (Figure S4). Increasing NH_4_OH also induced a relatively minor increase in the reaction rate (Figures S3 and S4) but correlated with strongly worsening morphology for the same reaction conditions, with the appearance of microscale pinholes by SEM (Figure S3) and a strongly hazy film appearance to the eye. This is likely caused by the known phenomenon that high amine concentrations can dissolve and liquefy perovskite and lead halide salts that then recrystallize.^29,30^ In this case, dewetting can occur if the surface tension is not engineered for optimal substrate wetting. Although this behavior was improved by reducing the amine concentration (Figure S3), it was still common to have some pinholes visible in the film (Figure 2c). With optimized reaction conditions, it was possible to achieve residual PbI_2_ diffraction signal 2–10× lower than perovskite signal as in Figure 2 and the other optimized devices in this work, which often results in optimal performance for perovskites formed by hybrid and two-step vapor reactions.^31,32^ At this point, proof of concept devices were fabricated but had low yield and reproducibility, with low open circuit voltage (*V*_*oc*_) and a high proportion of shunted devices, particularly once the films reached near full conversion.

Despite the initial difficulties in device fabrication, the optoelectronic properties of the films with optimized conversion and morphology were probed to see whether the material is otherwise suitable for photovoltaic applications. Optical spectroscopy was used to measure the reflection, transmission, and absorption of the perovskite. A Tauc plot to the corrected absorbance yields an optical bandgap of 1.55 eV (Figure 2d). Photoluminescence quantum yield (PLQY) measurements were performed to understand the balance between radiative and nonradiative recombination at one-sun-equivalent laser irradiance and confirm the bandgap. Figure 2e shows a typical spectrum, exhibiting a PLQY of over 0.1% with a peak center at 1.57 eV, showing that the perovskite has relatively good radiative efficiency despite the poor current–voltage (*J*–*V*) characteristics of initial solar cells.

The *J*–*V* curve of the champion device using NH_4_OH with MeO-2PACz SAM HTLs alone is shown in Figure 3b. Under the assumption that the poor morphology of the perovskite films contributes to the poor and irreproducible device performance observed in early devices, we tested whether the application of SiO_*x*_ nanoparticles on the MeO-2PACz surface could improve film formation.^33^ Solar cells using SiO_*x*_ nanoparticles on MeO-2PACz had improved morphology (Figure S7a) and higher shunt resistance. The champion device shown in Figure 3b had 146 mV higher *V*_*oc*_ and notably improved *V*_*oc*_ consistency across the whole batch (Figure S7b). Furthermore, the addition of NH_4_OH as an ammonia source was found to lead to some irreproducibility, presumably because NH_3_ is highly volatile even at room temperature and ambient pressure. This means the exact timing of dispensing the NH_4_OH and placing it in the reaction environment is critical to reproducibility and is difficult to control by hand. One solution to this problem on the lab scale is to use NH_4_oAC, which is a volatile solid that can be more reproducibly added in exact quantities and should only begin to release substantial quantities of ammonia upon heating. When using NH_4_oAC without nanoparticle addition, the films also showed improved α phase purity and nearly complete conversion (Figure 3a) but were still prone to shunting (Figure 3b) due to poor morphology (Figure S7a). By incorporating both nanoparticles and NH_4_oAC, a solar cell with 7.1% photon conversion efficiency (*PCE*) from the reverse scan was achieved with short circuit current (*J*_*sc*_) = 12.2 mA/cm^2^, *V*_*oc*_ = 935 mV, and fill factor (*FF*) = 62.1%. However, an intense single diffraction peak at 2θ = 23.7° appeared in the X-ray diffractogram, corresponding to a highly oriented 6H polytype FAPbI_3_ ^28^ (Figure 3a).

**Figure 3:**
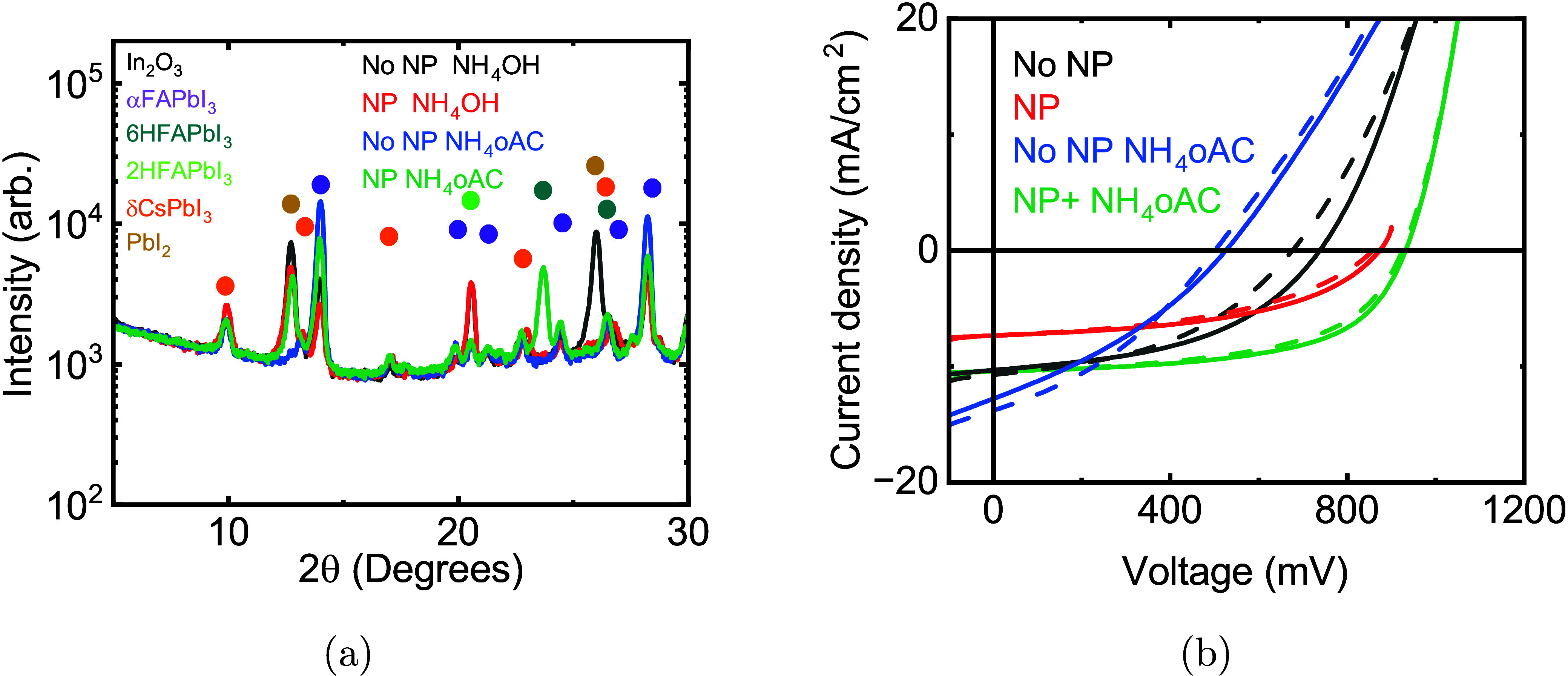
(a) Logarithmic X-ray diffraction patterns of films using NH_4_OH or NH_4_oAC and MeO-2PACz or MeO2-PACz/nanoparticle substrates. (b) The *J*–*V* curve of champion solar cells using the same hole transport layer and ammonia sources as in (a).

We hypothesized that because this peak is highly oriented and only appears when SiO_*x*_ nanoparticles are used to decorate the MeO-2PACz, they may be templated by this specific substrate and be suppressed by the use of a different HTL stack. Indeed, the strong and highly oriented 6H FAPbI_3_ peak was suppressed by the use of NiO_*x*_ nanoparticles instead of MeO-2PACz/SiO_*x*_ (Figure S9), but other, weaker hexagonal polytype peaks with different orientations were still observed. The use of a NiO_*x*_ nanoparticle HTL yielded a champion solar cell with similar *J*–*V* characteristics, with a slightly higher *PCE* from the reverse scan (7.5%), but a slightly lower stabilized *PCE* (Figure S10). This indicates that while the overall δ-phase and hexagonal polytype phase inclusions may be problematic for performance, this highly oriented phase alone is not the main factor limiting device efficiency.

Finally, a piperazinium iodide (PI) treatment was applied to solar cells to reduce nonradiative losses originating from the C_60_-perovskite interface.^34,35^ The champion solar cell with a structure glass/ITO/MEO-2PACz/SiO_*x*_ nanoparticles/FA_*x*_Cs_1–*x*_Pb­(I_*y*_Br_1–*y*_)_3_/PI/C_60_/SnO_*x*_/Ag was characterized (Figure 4). The champion solar cell reached 10.6% efficiency from a reverse *J*–*V* scan with little hysteresis. The stabilized power output was 10.3% and the device was stable under maximum power point tracking for more than 10 min in ambient air with no encapsulation and no cooling (Figure 4b). The FF of 73% and *V*_*oc*_ of 1.03 V is comparable to many other reports of two-step vapor-deposited perovskite solar cells.^32,36–42^ Figure 4c shows the external quantum efficiency (EQE) of the solar cell, which has a maximum EQE of only 60%. The integrated current from the EQE matches the *J*_*sc*_ from the *J*–*V* scan. The low *J*_*sc*_ of 14.1 mA/cm^2^ is a major limitation of these solar cells. The planar ITO substrates without any antireflection coating account for ca. 5–10% reduction due to reflection and parasitic absorption of the ITO and can be optimized using various strategies,^43^ leaving ca. 30% current collection losses likely driven by the notable δ-phase Cs and hexagonal polytype FAPbI_3_ phase inclusions as seen in Figure 4d, indicating poor mixing of FA and Cs phases. This is corroborated by the fact that peak EQE can be improved to 80% by applying reverse bias of up to −1.75 eV (Figure S8), showing that there are unresolved carrier collection issues. For a bandgap of 1.55 eV, improving *J*_*sc*_ to 20–23 mA/cm^2^ would yield 15–17% *PCE*. Further efforts to improve solar cell performance from this process will include a detailed study isolating and understanding the sources of loss in the solar cell, as well as implementing strategies to improve film uniformity, phase mixing, and phase purity.

**Figure 4:**
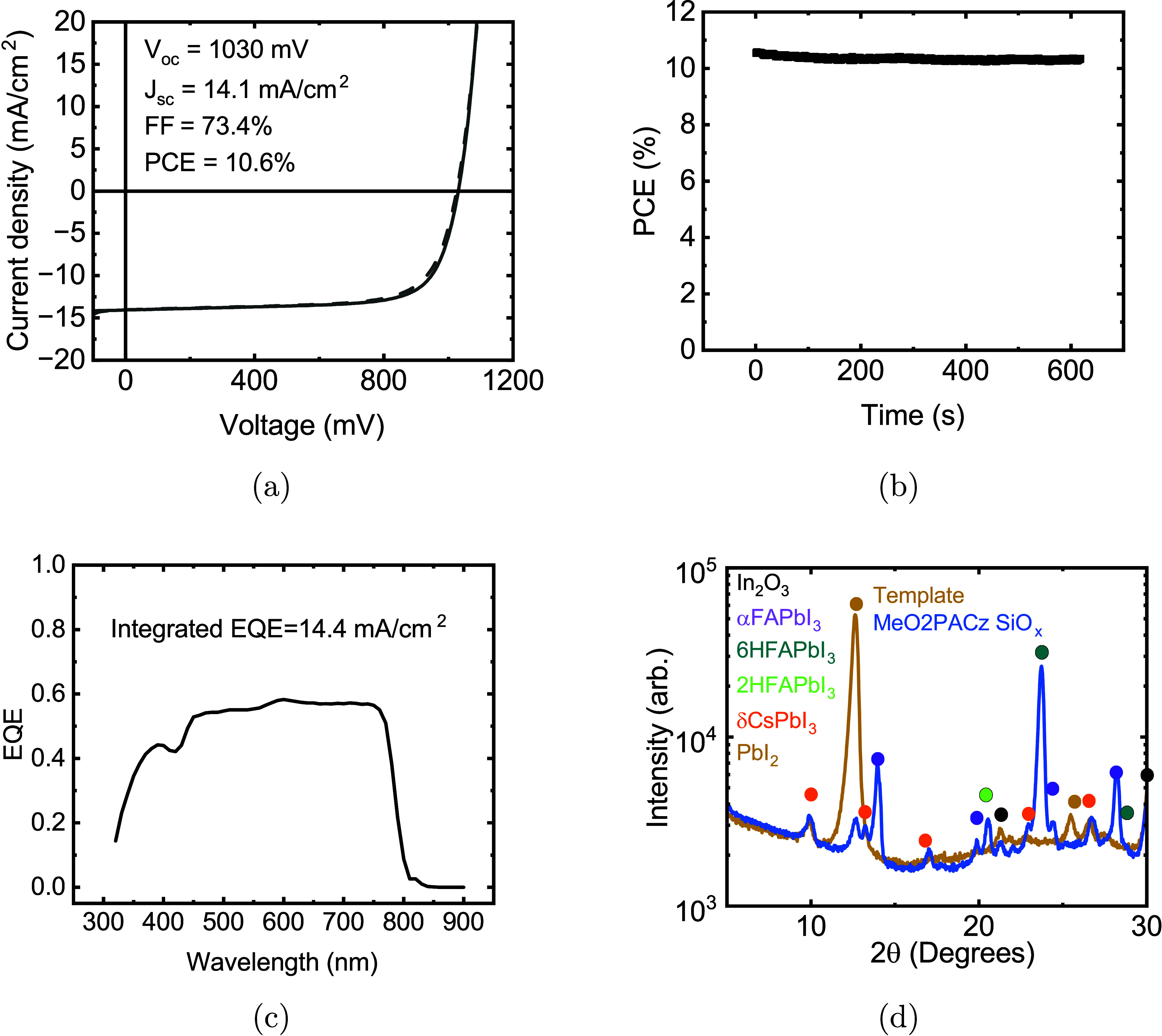
(a) The champion *J*–*V* curve of a solar cell with the structure glass/ITO/MeO-2PACz/SiO_*x*_ nanoparticles/FA_*x*_Cs_1–*x*_Pb­(I_*y*_Br_1–*y*_)_3_/C_60_/SnO_*x*_/Ag. (b) Maximum power point tracking of the same cell with a stabilized power output of 10.3%, stable for more than 10 min of unencapsulated operation in ambient air with no cooling. (c) EQE of the same cell showing a maximum EQE of approximately 60% and an integrated EQE current of 14.4 mA/cm^2^. (d) A log scale X-ray diffractogram of the same cell after measurement and a CsBr/PbI_2_ template produced in the same evaporation run to show template and substrate peaks.

Here we note that in this process, the vapor is transported over a distance of 10 cm at atmospheric pressure through ambient air with uncontrolled humidity before the conversion of the template to the perovskite occurs. All of the ingredients have boiling points of ≤ 127 °C and s-triazine and ammonia do not incorporate into the perovskite at the optimal reaction conditions. This lends itself to the possibility that this new chemical pathway can be used in vapor-transport-like processes. Indeed, the current small batch process is limited in throughput and precision of control of the reactants, with milligram inaccuracies in weighing contributing to large percentage changes in the reactor dose (e.g., 2 mg NH_4_oAC leads to a 5% change in concentration assuming all powder is evaporated). This work also makes it evident that the conversion, morphology, and phase composition are all sensitive to the exact conditions and ratios of ingredients provided to the reactor. Thus, in future iterations of this work we envision a vapor-transport like process with improved control that will enable better control of the reactants and reaction conditions. This would entail: precise control of the substrate temperature, mass flow control of the reactants into the reaction environment, improved vapor mixing through showerhead or baffle design, and control of the reaction atmosphere with inert, dry air, or controlled humidity. Further improvements will require precise tuning of the reaction and the achievement of even better compositional control and phase purity. Substrate and template engineering, as well as additives, should be explored.

In conclusion, in this work we show an entirely new chemical pathway for ambient-pressure in-air vapor-phase deposition of FA-rich perovskite solar cells, where chemicals that are often thought of as chemical decomposition products of formamidinium halide evaporation (i.e., s-triazine, ammonia, and HI) are used to create formamidine in situ and eventually form a perovskite phase. The formamidine-rich composition was verified by NMR and the presence of α.phase perovskite was verified by XRD. Proof-of-concept solar cells attained >10% *PCE*. The reaction was complete within 15 min, which may be able to be accelerated by improving the control of the geometry, concentration, and heat profile of the reactions in future iterations. It is expected that optimization of film morphology and phase purity will yield pathways to more competitive efficiencies. These components are highly volatile, cheap, and easy to evaporate: thus, we envision this process may be applied to vapor transport deposition processes and help create new high-rate industry-compatible perovskite deposition methods.

## Supplementary Material



## Notes

Experimental details; pictures of the reaction vessel; X-ray diffraction for the series altering the addition of each reactant; SEM images of films with different ammonia sources and SAM or NP/SAM HTL stacks; bias dependence of EQE; *J*–*V* curve, maximum power point tracking, and XRD for solar cells produced on NiO_*x*_ nanoparticles (PDF)

## References

[1] NREL Photovoltaic Research Best Research-Cell Efficiency Chart. 2024; https://www.nrel.gov/pv/cell-efficiency.html, Accessed: 2024–12–08.

[2] Grancini G., Roldan-Carmona C., Zimmermann I., Mosconi E., Lee X., Martineau D., Narbey S., Oswald F., De Angelis F., Graetzel M., Nazeeruddin M. K. (2017). One-Year stable perovskite solar
cells by 2D/3D interface engineering. Nat. Commun..

[3] Christians J. A., Schulz P., Tinkham J. S., Schloemer T. H., Harvey S. P., Tremolet de Villers B.
J., Sellinger A., Berry J. J., Luther J. M. (2018). Tailored interfaces of unencapsulated
perovskite solar cells for > 1,000 h operational stability. Nat. Energy.

[4] Azmi R. (2022). Damp heat–stable perovskite solar cells with
tailored-dimensionality
2D/3D heterojunctions. Science.

[5] Zhao Y. (2022). A bilayer conducting
polymer structure for planar perovskite solar
cells with over 1,400 h operational stability at elevated temperatures. Nat. Energy.

[6] Suo J. (2024). Multifunctional sulfonium-based treatment for perovskite
solar cells
with less than 1% efficiency loss over 4,500-h operational stability
tests. Nat. Energy.

[7] Sidhik S. (2024). Two-dimensional perovskite
templates for durable, efficient formamidinium
perovskite solar cells. Science.

[8] Yoo J. J., Seo G., Chua M. R., Park T. G., Lu Y., Rotermund F., Kim Y. K., Moon C. S., Jeon N. J., Correa-Baena J. P., Bulović V., Shin S. S., Bawendi M. G., Seo J. (2021). Efficient
perovskite solar cells via improved carrier management. Nature.

[9] Park J., Kim J., Yun H. S., Paik M. J., Noh E., Mun H. J., Kim M. G., Shin T. J., Seok S. I. (2023). Controlled
growth
of perovskite layers with volatile alkylammonium chlorides. Nature.

[10] Chen H. (2024). Improved charge extraction in inverted perovskite solar
cells with
dual-site-binding ligands. Science.

[11] Dewi H. A., Li J., Wang H., Chaudhary B., Mathews N., Mhaisalkar S., Bruno A. (2021). Excellent Intrinsic Long-Term Thermal Stability of Co-Evaporated
MAPbI3 Solar Cells at 85 ^°^C. Adv. Funct. Mater..

[12] Li J., Wang H., Chin X. Y., Dewi H. A., Vergeer K., Goh T. W., Lim J. W. M., Lew J. H., Loh K. P., Soci C., Sum T. C., Bolink H. J., Mathews N., Mhaisalkar S., Bruno A. (2020). Highly Efficient Thermally Co-evaporated
Perovskite Solar Cells and Mini-modules. Joule.

[13] Abzieher T., Moore D. T., Roß M., Albrecht S., Silvia J., Tan H., Jeangros Q., Ballif C., Hoerantner M. T., Kim B.-S. (2024). Vapor
phase deposition of perovskite photovoltaics:
Short track to commercialization?. Energy Environ.
Sci..

[14] Wang S., Tan L., Zhou J., Li M., Zhao X., Li H., Tress W., Ding L., Graetzel M., Yi C. (2022). Over 24% efficient
MA-free Cs_x_FA_1-x_PbX_3_ perovskite solar
cells. Joule.

[15] Li H., Zhou J., Tan L., Li M., Jiang C., Wang S., Zhao X., Liu Y., Zhang Y., Ye Y., Tress W., Yi C. (2022). Sequential
vacuum-evaporated perovskite
solar cells with more than 24% efficiency. Sci.
Adv..

[16] Zhou J., Tan L., Liu Y., Li H., Liu X., Li M., Wang S., Zhang Y., Jiang C., Hua R., Tress W., Meloni S., Yi C. (2024). Highly efficient and
stable perovskite solar cells via a multifunctional hole transporting
material. Joule.

[17] Latini A., Gigli G., Ciccioli A. (2017). A study on
the nature of the thermal
decomposition of methylammonium lead iodide perovskite, CH3nh3PBI3:
An attempt to rationalise contradictory experimental results. Sustainable Energy Fuels.

[18] Baekbo M. J., Hansen O., Chorkendorff I., Vesborg P. C. (2018). Deposition of methylammonium
iodideviaevaporation–combined kinetic and Mass Spectrometric
Study. RSC Adv..

[19] Raga S. R., Ono L. K., Qi Y. (2016). Rapid perovskite
formation by CH_3_NH_2_ gas-induced intercalation
and reaction of PbI_2_. J. Mater. Chem.
A.

[20] Grundmann C., Kreutzberger A. (1955). The Ring Cleavage
of s-Triazine by Primary Amines.
A New Method for the Synthesis of Hererocycles. J. Am. Chem. Soc..

[21] Juarez-Perez E. J., Ono L. K., Qi Y. (2019). Thermal degradation of formamidinium
based lead halide perovskites into: Sym -triazine and hydrogen cyanide
observed by coupled thermogravimetry-mass spectrometry analysis. J. Mater. Chem. A.

[22] Thampy S., Zhang B., Park J. G., Hong K. H., Hsu J. W. (2020). Bulk and
interfacial decomposition of formamidinium iodide (HC­(NH_2_)_2_I) in contact with metal oxide. Mater. Adv..

[23] Sahli, F. Development of Highly Efficient Perovskite-on-Silicon Tandem Solar Cells. Ph.D. thesis, EPFL, 2020.

[24] Sahli, F. ; Guesnay, Q. ; Salsi, N. ; Duchêne, L. ; Ballif, C. ; Jeangros, Q. Ammonia-assisted vapour transport deposition of formamidinium salts for perovskite thin films; 2021; https://infoscience.epfl.ch/handle/20.500.14299/180825, Preprint, Accessed: 2022–10–14.

[25] Kroll M., Öz S. D., Zhang Z., Ji R., Schramm T., Antrack T., Vaynzof Y., Olthof S., Leo K. (2022). Insights into
the evaporation behaviour of FAI: material degradation and consequences
for perovskite solar cells. Sustainable Energy
Fuels.

[26] Grundmann C., Ratz R. (1956). Triazines.
XVI. A new synthesis for 1, 2, 4-triazoles. J. Org. Chem..

[27] Stoumpos C. C., Malliakas C. D., Kanatzidis M. G. (2013). Semiconducting tin and lead iodide
perovskites with organic cations: Phase transitions, high mobilities,
and near-infrared photoluminescent properties. Inorg. Chem..

[28] Gratia P., Zimmermann I., Schouwink P., Yum J.-H., Audinot J.-N., Sivula K., Wirtz T., Nazeeruddin M. K. (2017). The Many
Faces of Mixed Ion Perovskites: Unraveling and Understanding the Crystallization
Process. ACS Energy Lett..

[29] Zhou Z., Wang Z., Zhou Y., Pang S., Wang D., Xu H., Liu Z., Padture N. P., Cui G. (2015). Methylamine-Gas-Induced
Defect-Healing Behavior of CH 3 NH 3 PbI 3 Thin Films for Perovskite
Solar Cells. Angew. Chem..

[30] Wang Y., Lv P., Pan J., Chen J., Liu X., Hu M., Wan L., Cao K., Liu B., Ku Z., Cheng Y. B., Lu J. (2023). Grain Boundary
Elimination via Recrystallization-Assisted Vapor Deposition
for Efficient and Stable Perovskite Solar Cells and Modules. Adv. Mater..

[31] Cao D. H., Stoumpos C. C., Malliakas C. D., Katz M. J., Farha O. K., Hupp J. T., Kanatzidis M. G. (2014). Remnant PbI_2_, an unforeseen
necessity in high-efficiency hybrid perovskite-based solar cells?. APL Mater..

[32] Kuba A. G., Harding A. J., Richardson R. J., McCandless B. E., Das U. K., Dobson K. D., Shafarman W. N. (2022). Two-Step
Close-Space Vapor Transport of MAPbI3Solar Cells: Effects of Electron
Transport Layers and Residual PbI_2_. ACS Appl. Energy Mater..

[33] Turkay D., Artuk K., Chin X.-Y., Jacobs D. A., Moon S.-J., Walter A., Mensi M., Andreatta G., Blondiaux N., Lai H., Fu F., Boccard M., Jeangros Q., Wolff C. M., Ballif C. (2024). Synergetic substrate
and additive engineering for over 30%-efficient perovskite-Si tandem
solar cells. Joule.

[34] Li F. (2020). Regulating Surface Termination
for Efficient Inverted Perovskite
Solar Cells with Greater Than 23% Efficiency. J. Am. Chem. Soc..

[35] Mariotti S. (2023). Interface engineering for high-performance, triple-halide perovskite-silicon
tandem solar cells. Science.

[36] Chen Q., Zhou H., Hong Z., Luo S., Duan H. S., Wang H. H., Liu Y., Li G., Yang Y. (2014). Planar heterojunction
perovskite solar cells via vapor-assisted solution process. J. Am. Chem. Soc..

[37] Guo Q., Li C., Qiao W., Ma S., Wang F., Zhang B., Hu L., Dai S., Tan Z. (2016). The growth of a CH_3_NH_3_PbI_3_ thin film using simplified close space sublimation
for efficient and large dimensional perovskite solar cells. Energy Environ. Sci..

[38] Ng A., Ren Z., Shen Q., Cheung S. H., Gokkaya H. C., So S. K., Djurišić A. B., Wan Y., Wu X., Surya C. (2016). Crystal Engineering for Low Defect Density and High Efficiency Hybrid
Chemical Vapor Deposition Grown Perovskite Solar Cells. ACS Appl. Mater. Interfaces.

[39] Hoerantner M. T., Wassweiler E. L., Zhang H., Panda A., Nasilowski M., Osherov A., Swartwout R., Driscoll A. E., Moody N. S., Bawendi M. G., Jensen K. F., Bulović V. (2019). High-Speed
Vapor Transport Deposition of Perovskite Thin Films. ACS Appl. Mater. Interfaces.

[40] Sahli F., Miaz N., Salsi N., Bucher C., Schafflützel A., Guesnay Q., Duchêne L., Niesen B., Ballif C., Jeangros Q. (2021). Vapor Transport Deposition
of Methylammonium Iodide
for Perovskite Solar Cells. ACS Appl. Energy
Mater..

[41] Guesnay Q. (2024). Pizza Oven Processing of Organohalide Perovskites (POPOP): A Simple,
Versatile and Efficient Vapor Deposition Method. Adv. Energy Mater..

[42] Rodkey N., Gomar-Fernández I., Ventosinos F., Roldan-Carmona C., Koster L. J., Bolink H. J. (2024). Close-space sublimation
as a scalable method for perovskite solar cells. ACS Energy Lett..

[43] Rodkey N., Zanoni K. P., Piot M., Dreessen C., Grote R., Carroy P., Sebastian Alonso J. E., Paliwal A., Muñoz D., Bolink H. J. (2024). Efficient micrometer
thick bifacial perovskite solar
cells. Adv. Energy Mater..

