# Supplementary material for: Making from Breaking: Degradation Inversion Enables Vapor-Phase Synthesis of Halide Perovskites in Ambient Conditions
Source: ACS Energy Lett. 2025 May 12;10(6):2710–7. doi: 10.1021/acsenergylett.4c03395 (PMC12172037; doi:10.1021/acsenergylett.4c03395)
Supplement: Supplementary file 1 [file nz4c03395_si_001.pdf]

Making from Breaking: Degradation Inversion Enables Vapor-Phase Perovskite Synthesis in Ambient Conditions

Austin Kuba[1], Florent Sahli[2], Mostafa Othman[1], Kerem Artuk[1], Quentin Jeangros[2], Aïcha Hessler-Wyser[1], Christophe Ballif[1], Christian M. Wolff[1]

[1] Ecole Polytechnique Fédérale de Lausanne (EPFL), Institute of Electrical and Microengineering (IEM), Photovoltaics and Thin-Film Electronics Laboratory, 2002 Neuchâtel, Switzerland

[2] Centre Suisse d'Electronique et de Microtechnique (CSEM), 2002 Neuchâtel Switzerland

Experimental details

Chemical suppliers:

PbI_2: Sigma Aldrich, 99.999% (beads)

CsBr: ABCR 99.999%

s-triazine: Sigma Aldrich, 97%

NH_4OH: Sigma Aldrich, ACS reagent, 28-30% NH_3 basis

NH_4oAC: Sigma Aldrich, ACS reagent, >97%

HI: Sigma Aldrich, stabilized, >47%

MEO2PACz: TCI Chemicals, >98%

Solar cell fabrication

ITO/glass substrates were sonicated in helmanex/deionized water (DI) solution for 10 minutes. The substrates were rinsed 5x in DI water and sonicated for 10 minutes in DI. Immediately before HTL deposition, the cleaned ITO substrates were exposed to a UV-ozone treatment for 15 minutes.

For MEO2PACz HTL, a solution of 1mg/ml MEO2PACz in ethanol was dispensed statically, with a 10 second delay before spinning at 3000 RPM for 30 seconds. With 20s to go, 115 µl MEO2pacz solution was dispensed dynamically on the substrate. The films were then annealed for 10 minutes at 100C. Next, the films were rinsed with ethanol. 115 µl ethanol was dispensed on the substrate with a 10s delay before spinning at 3000 rpm for 30 seconds. 115 µl ethanol was dispensed on the substrate with 20s remaining. For the films with SiO_x nanoparticles, 1 wt% 25 nm SiO_x nanoparticle dispersion in ethanol was further diluted 5:1 with ethanol:NP solution. 115 µl of diluted SiO_x nanoparticle solution was dispensed statically with a 10s delay before spinning at 3000 RPM for 30 seconds. The films were dried for 10 minutes at 100C.

For NiO_x nanoparticle HTL, 5 mg/mL of NiO_x nanoparticles were dissolved in a 3:1 DI/IPA solvent mixture. 115 µl solution was dispensed statically before ramping to 3000 RPM for 30s. The films were annealed at 125C for 10 minutes.

Lead-halide alkali-halide templates were coevaporated in a Lesker minispectros evaporator. 300 nm PbI_2 was coevaporated with 30 nm of CsBr at rates of 1 and 0.1 A/s, respectively. The base pressure was $<1.5x10^{-6}$ mTorr.

The organohalide conversion step was carried out in a fume hood using a staining glass (Figure S1). The staining glass was preheated at 150C for 30 minutes. Vials with different amounts of s-triazine, an ammonia source such as NH_4OH solution or NH_4oAC, and HI were prepared. A template was taped to the lid of the staining glass and preheated for 5 minutes. The vials were placed quickly into the staining glass and the lid with the template was placed on top of the jar. The reaction was allowed to proceed for 9-15 minutes until the film turned brown to the eye. If the reaction was allowed to proceed further, the films would turn transparent (as is known to occur for treatments with ammonia and methylamine [1,2]), whereupon removing the lid would sometimes turn the film brown again and annealing at 150C also caused the films to turn brown again. After each reaction, every film was annealed at 150C for 1 minute. Between reactions, to reset the reaction environment, the jar was allowed to rest on the hotplate with no lid, and blown with N_2 gas to allow all condensed liquids to evaporate. The condition which gave the best condition across multiple substrates was 150C hotplate, 15-minute reaction time, 80 mg s-triazine, 60 µl HI, 40 mg NH_4oAC (or 100 µL NH_4OH). The addition amounts need to be adjusted to the size of the reaction environment.

Piperazinium iodide interface passivation: for some cells, piperazinium iodide solution (0.3 mg/ml in IPA) was dynamically dispensed on the film surface at 5000 rpm and spun for 30s followed by 5 minutes of annealing at 100C.

To complete the cell, 30 nm of C_{60} was evaporated in a custom-built evaporator at a rate of 0.3 Angstrom/s. Next, 225 cycles of ALD SnO_x was deposited on the C_{60} with a substrate temperature of 90C using a PICOSUN R-200 Standard ALD system. Finally, 100 nm of Ag was evaporated in a custom-built evaporator at a rate of 0.3 Angstrom/s to serve as a back electrode.

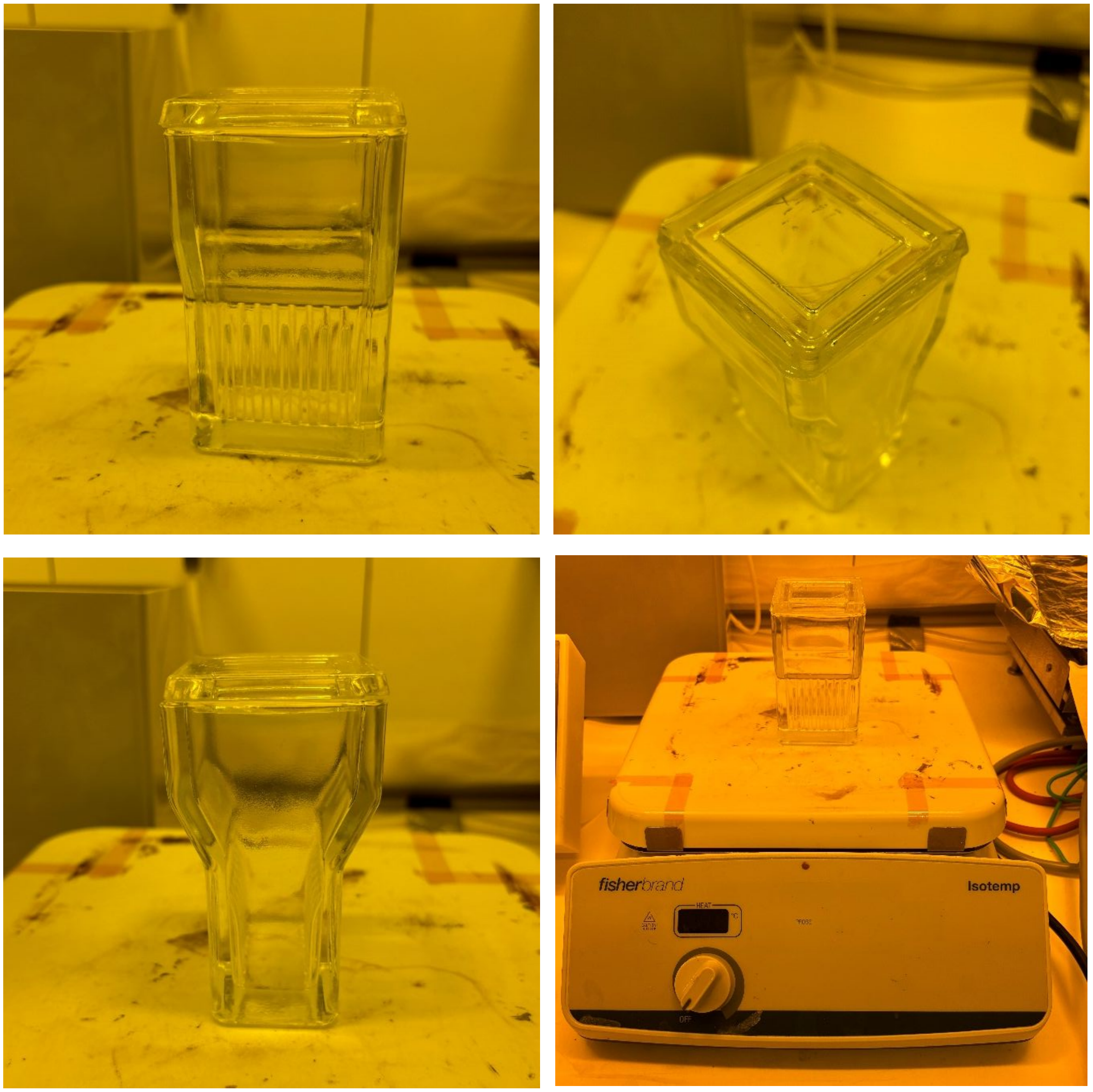

Figure S1. Different perspectives of the staining glass and the hotplate setup.

Characterization

1H NMR spectra were recorded on a Bruker AV4 spectrometer (1H: 400.1 MHz) equipped with a 5 mm BBO probe at 298 K. Spectra were acquired with a 30° pulse of 3.3 µs, a spectral width of 7.8 kHz (20 ppm), an acquisition time (aq) of 4 s, a preparation delay (d1) of 1 s, and between 16 and 128 accumulations. The free induction decays were zero-filled to 65 K points and multiplied by a 0.3 Hz exponential line-broadening function prior to Fourier transformation to the frequency domain. 1H Chemical shifts were referenced to TMS using the signals of the residual protons of the deuterated solvent (δ1H = 2.50 ppm (DMSO-d6)) as secondary reference.

UV-vis measurements were performed with a PerkinElmer Lambda 900 UV-Vis-NIR spectrophotometer fitted with an integrating sphere.

Photoluminescence quantum yield and photoluminescence spectra were measured with a homemade system. A 532 nm laser is input into an integrating sphere with a fiber optic cable. The laser was calibrated with a photodetector to deliver the same photon flux as would be absorbed under 1 sun light for a bandgap of 1.6eV. The laser shines onto the sample. An Ocean Optics USB2000 detector and Ocean Insight Maya2000 Pro detector collect sample emission and laser excitation intensities, respectively, using a bifurcated fiber from ThorLabs. As a calibration check, three fluorescent test samples with high specified PLQY (≈70%) supplied from Hamamatsu Photonics were measured where the specified value could be accurately reproduced within a small relative error of less than 5%. For each measurement run a dark calibration (laser blocked by the shutter, integrating sphere closed) and a light calibration (laser incident to integrating sphere with no sample in the sphere) was taken to understand the noise floor and to calibrate the laser absorbance. The typical integration times were 1s for the Maya2000 Pro and 30 ms for the USB200 detector.

X-ray diffraction was performed in Bragg Brentano geometry with a copper Kα source with a Kβ filter using a PANalytical Xpert Pro MPD.

Current density voltage curves are measured with a class AAA WACOM solar simulator calibrated to 1000W/m^2 illumination intensity. Shadow masks are used to define the illuminated solar cell area to 0.1 cm^2. The measurements are done in ambient air without cooling.

The external quantum yield of perovskite solar cells was measured with a custom-made spectral response set-up where the samples were irradiated with chopped light at a frequency of 217 Hz and the response measured with a lock-in amplifier.

SEM images were taken using a Zeiss Gemini 2 microscope using a secondary electron detector and an acceleration voltage of 3 kV.

Supporting Figures:

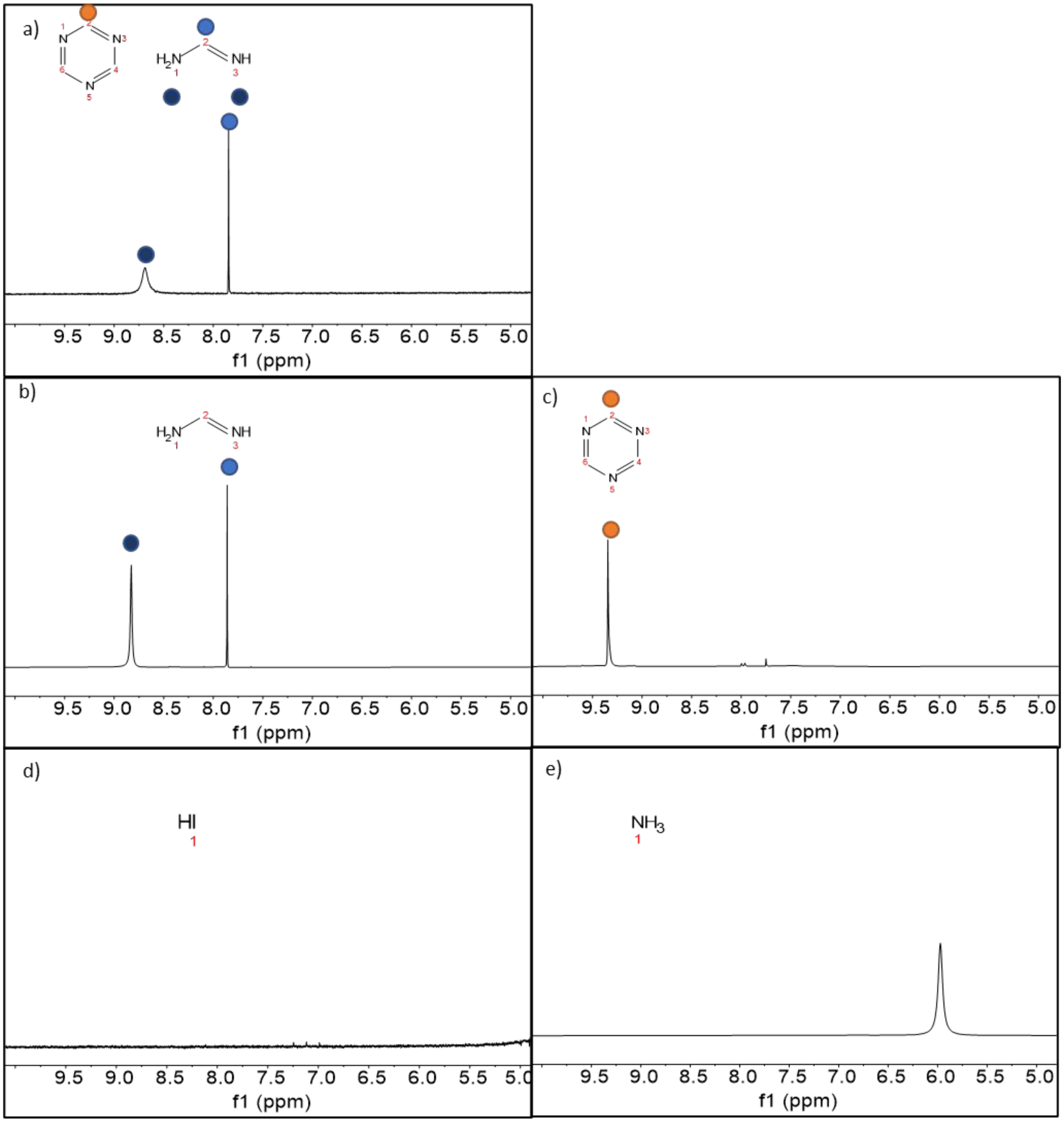

Figure S2. 1H NMR of a) six perovskite thin films produced by the s-triazine method, b) FAI, c) s-triazine, d) HI, e) NH_4oAC all dissolved in DMSO-d6.

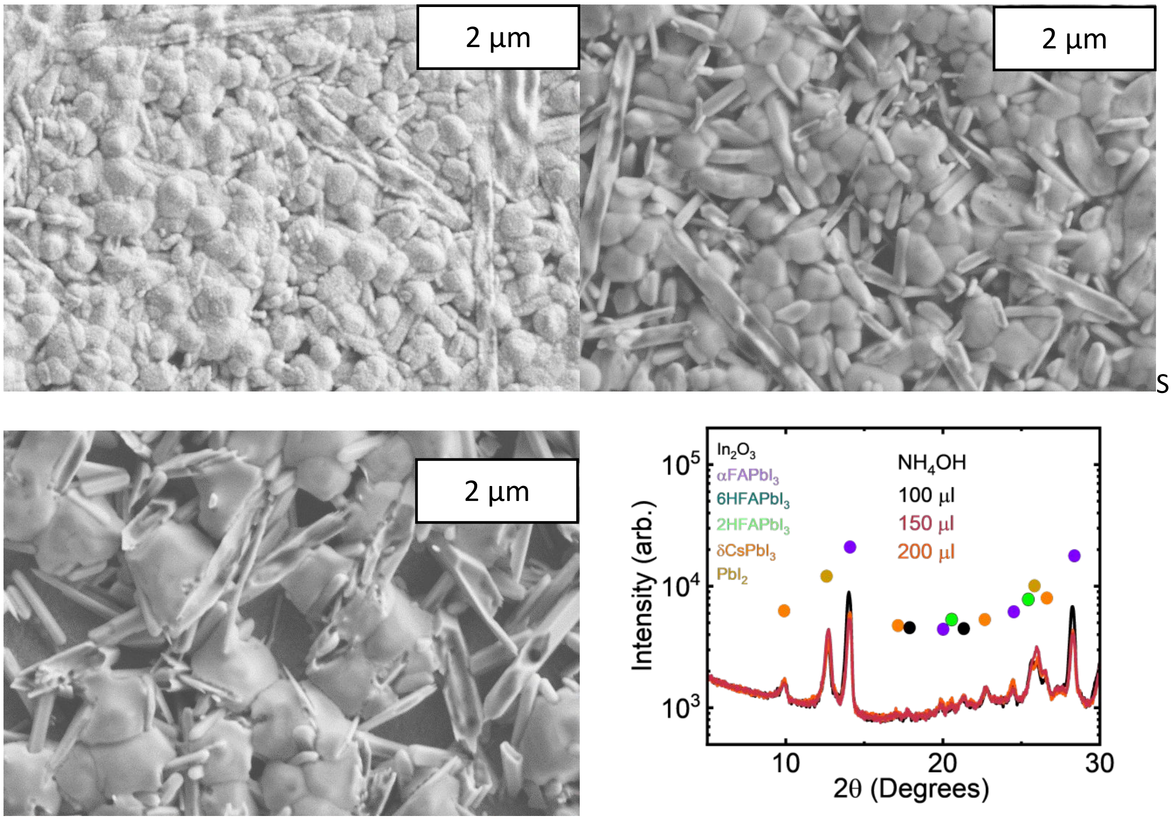

Figure S3. SEM of films with increasing NH_4OH content added to the reaction. The XRD shows that the films have similar phase compositions, but the SEM shows that increasing ammonia causes coarsening grains with pinholes appearing.

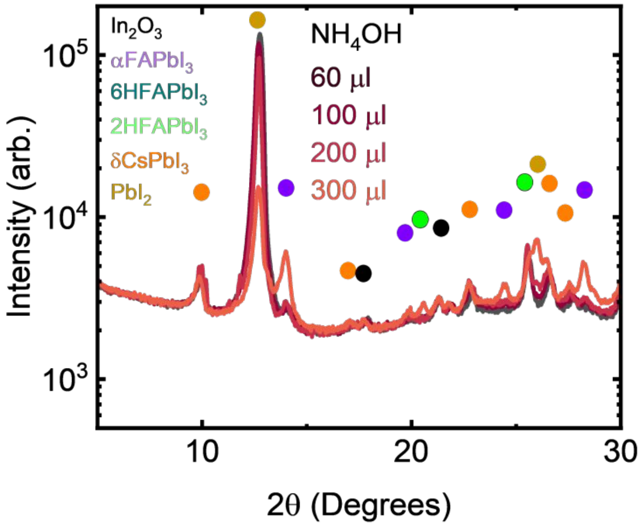

Figure S4. A series of films made with increasing NH_4OH concentration. Between 60 and 200 microliters of NH4OH addition, there is a minor effect on conversion. Further addition to 300 microliters does appear to make a more substantial effect on conversion but this is already beyond the point where excessively poor morphologies are observed (Figure S1.)

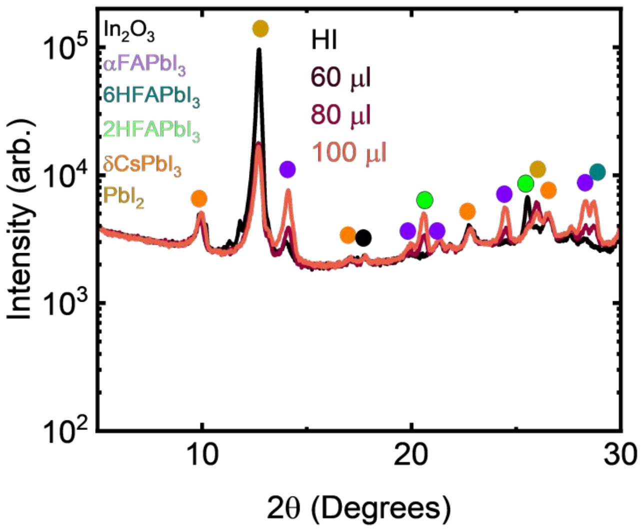

Figure S5. A series of films made with increasing HI addition. Increasing HI from 60 to 100 μl of HI leads to increase of perovskite intensity but also leads to a notable increase in hexagonal FAPbI3 polytype diffraction signals.

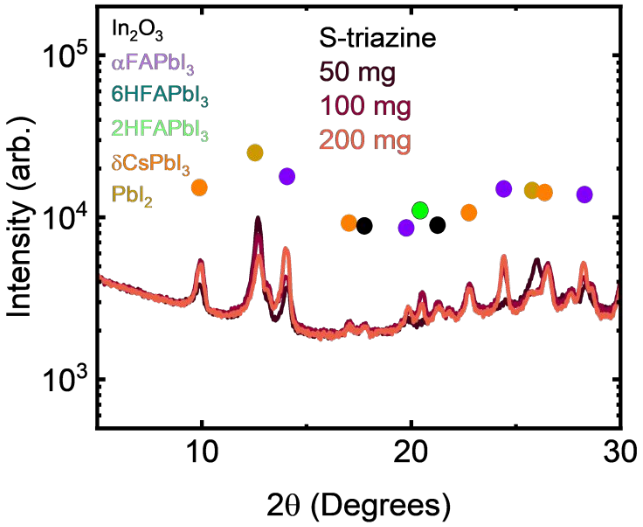

Figure S6. Increasing s-triazine addition increases the conversion slightly without creating major differences in phase composition.

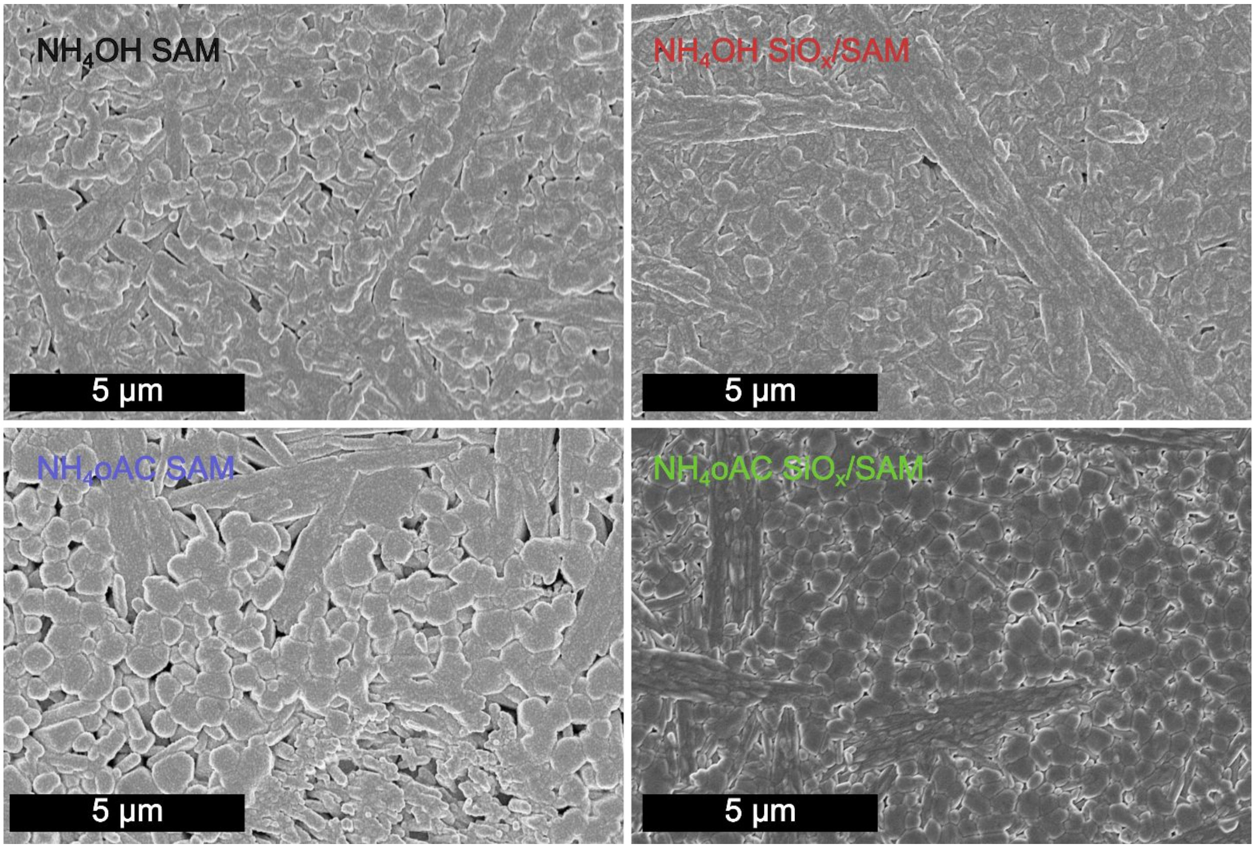

Figure S7a. SEM images of the champion devices from Figure 3a and 3b. The use of NH_4oAC leads to larger domains but more pin-hole like formation, while the use of nanoparticles consistently improves the morphology, with smaller pinholes and lower density of pinholes.

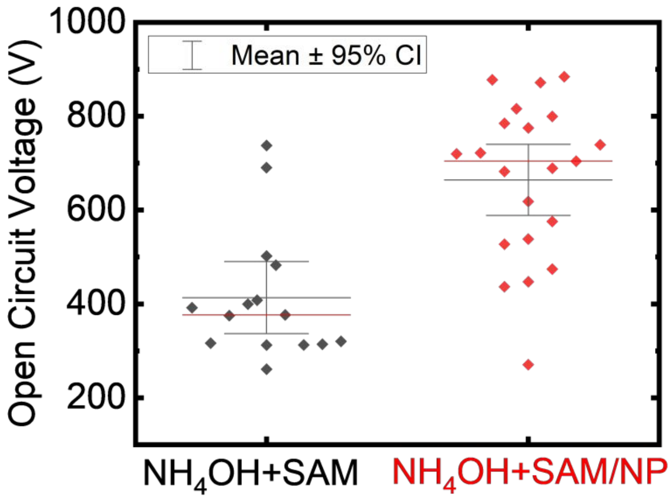

Figure S7b. V_{oc} statistics of devices using NH_4OH with SAM or SAM/SiO_x nanoparticle HTLs. The nanoparticles improve average V_{oc} as evidenced by the nonoverlapping 95% confidence interval.

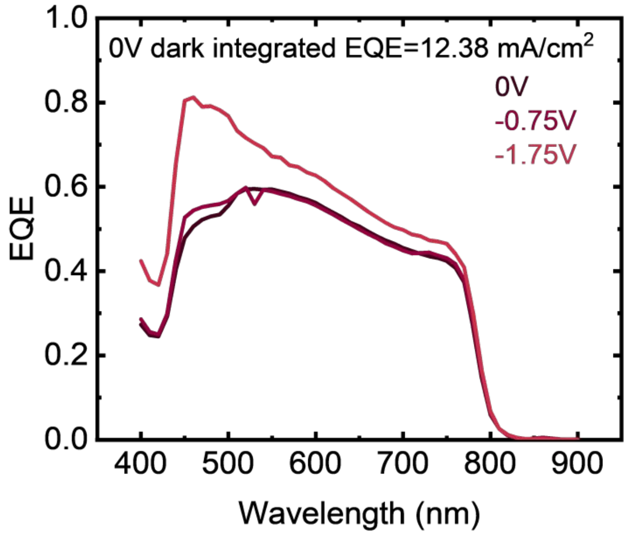

Figure S8. EQE of a film on MEO2PACz with SiO_x nanoparticles using NH_4oAC as the ammonia source. It originally has a low peak EQE and incomplete collection at long wavelengths. PI passivation improves collection at long wavelengths (Figure 4c) implicating poor collection near the C_{60} interface. Reverse bias of -1.75V increases the peak EQE to 80%, indicating charge extraction issues in the solar cells that need to be overcome to improve current collection. We expect this is caused by the high delta phase incorporation.

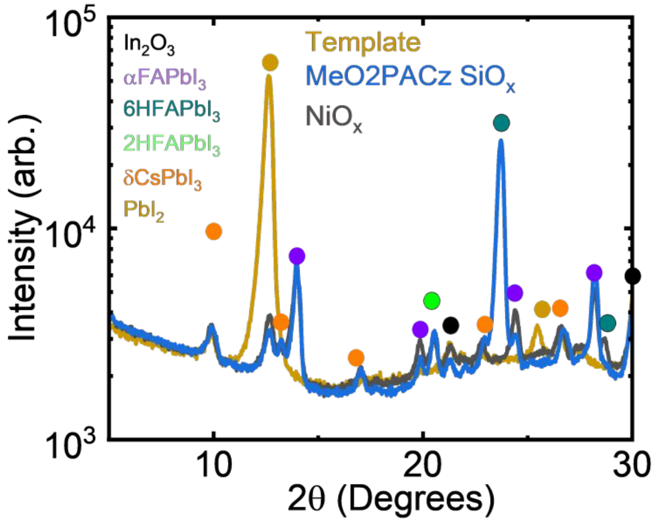

Figure S9. The devices using NiO_x nanoparticles show a notable suppression in the intense delta phase peak at 23.7 degrees, although some delta phase Cs and FA peaks persist.

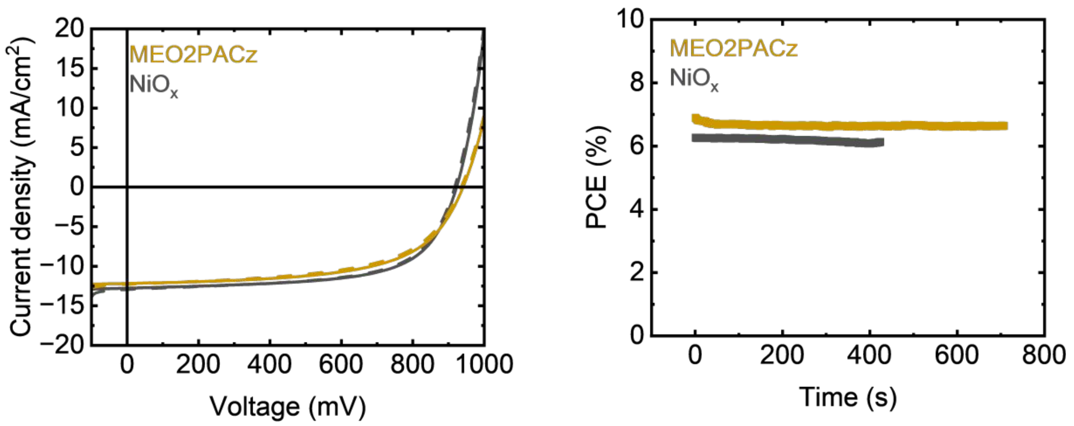

Figure S10. The use of NiO_x instead of MEO2PACz as an HTL lead to minimal overall changes in device behavior. Using NiO_x the PCE from the JV scan is slightly higher but from maximum power point tracking it is slightly lower.

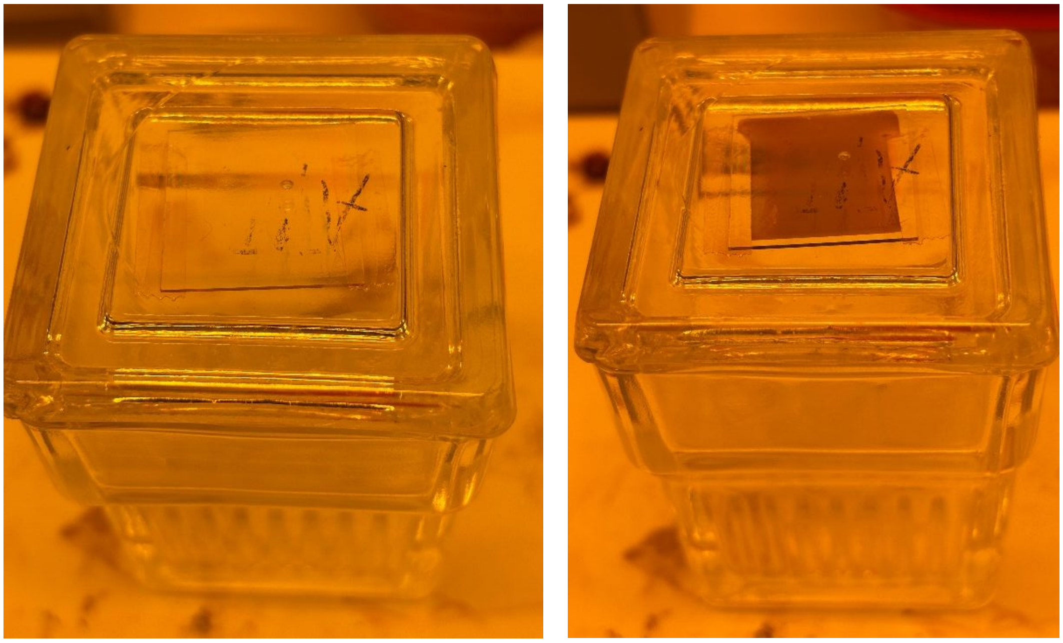

Figure S11. A picture of film in the reaction environment at <1 minutes and after 15 minutes of reaction at 150C with optimized reaction conditions

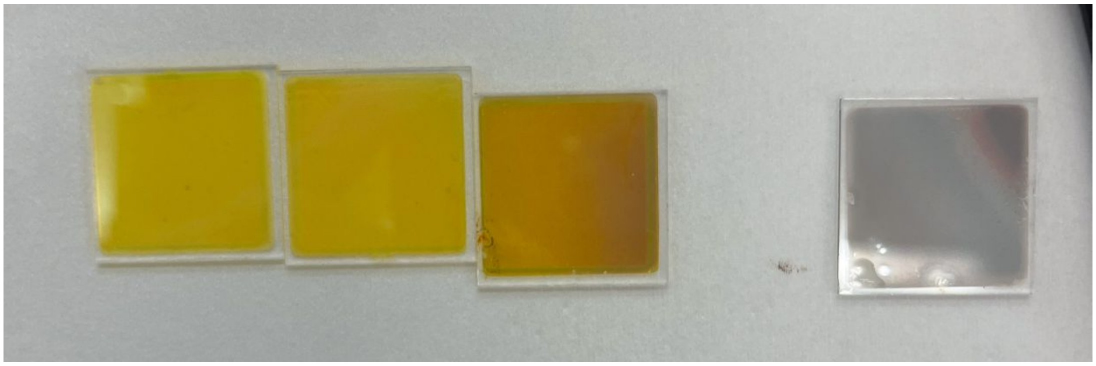

Figure S12a. The first films using I_2 as a iodide source. The jar was dosed with 80 mg s-triazine, 100 µl NH_4OH, and two beads of solid I_2 for each reaction. For these films, the lid was not preheated. Film 1 (left) used 60C 10 minutes, film 2 used 100C 10 minutes, film 3 used 150C 10 minutes, and film 4 used 150C 30 minutes. The films using I_2 as a halide source show less homogeneity from left to right (as in the case of a more line of sight evaporation) than HI samples. It also is difficult to precisely dose the iodide using irregularly shaped solid iodide beads. This problem may be solved in the future by engineering a dedicated system supplying the vapor in a controlled quantity and homogenizing the vapor before it arrives at the substrates.

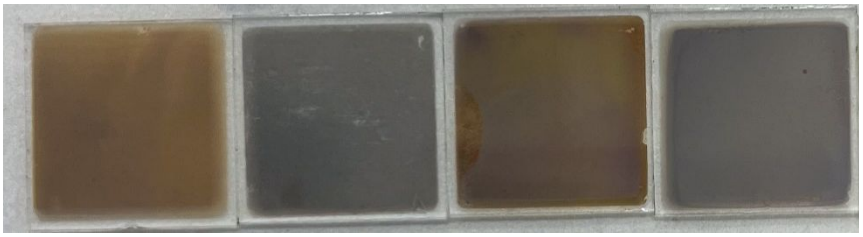

Figure S12b. The second set of films using I_2 as a vapor source. The jar was dosed with 80 mg s-triazine, 100 ul NH_4OH, and two beads of solid I_2 for each reaction. Film one (left) used 150C with a cold lid and 10 minutes reaction. Film two used a cold lid and 15 minutes of reaction. Film three used a preheated lid and 20 minutes of reaction. Film 4 used a preheated lid and 25 minutes of reaction. This shows that the lid preheating changes the speed of the reaction (with a hot lid slowing the reaction due to lower adsorption of species onto the film). A hot lid was chosen for future runs as sample 3 had the smoothest and most uniform morphology to the eye. Films that are reacted to the point where they turn completely black as in films 2 and 4 have no residual PbI_2 but have a hazy appearance indicating rough morphology that is problematic for devices. This indicates that I_2 has potential for use in this process but better control of dosing is required.

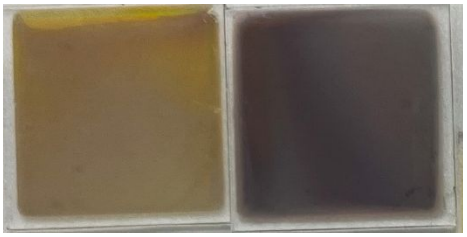

Figure S12c. The first set of films using HI as a vapor source. The jar was dosed with 80 mg s-triazine, 100 ul NH_4OH, and 100 µl of HI for each reaction. Film one (left) used 20 minutes with 150C. Film two used 25 minutes with 150C. The optimized film has a more homogenous and smooth appearance than the

films using I_2 as a source, and the repeatability of HI liquid dosing was better than solid iodine bead dosing, so HI was chosen for further trials.

References:

[1] Zhou, Z.; Wang, Z.; Zhou, Y.; Pang, S.; Wang, D.; Xu, H.; Liu, Z.; Padture, N. P.; Cui, G. Methylamine-Gas-Induced Defect-Healing Behavior of CH 3 NH 3 PbI 3 Thin Films for Perovskite Solar Cells. Angewandte Chemie 2015, 127, 9841–9845.

[2] Wang, Y.; Lv, P.; Pan, J.; Chen, J.; Liu, X.; Hu, M.; Wan, L.; Cao, K.; Liu, B.; Ku, Z.; Cheng, Y. B.; Lu, J. Grain Boundary Elimination via Recrystallization-Assisted Vapor Deposition for Efficient and Stable Perovskite Solar Cells and Modules. Advanced Materials 2023, 35, 2304625.
